# Supplementary material for: Neurons in primary auditory cortex represent sound source location in a cue-invariant manner
Source: Nat Commun. 2019 Jul 9;10:3019. doi: 10.1038/s41467-019-10868-9 (PMC6616358; doi:10.1038/s41467-019-10868-9)
Supplement: Supplementary file 1 — Supplementary information [file 41467_2019_10868_MOESM1_ESM.pdf]

Neurons in primary auditory cortex represent sound source location in a cue-invariant manner

Wood et al.

	Ferret			
Stimulus	F1301	F1302	F1310	F1313
BBN	865 / 1196<br>72.3%<br>$p < 0.001$	2726 / 3845<br>70.9%<br>$p < 0.001$	1557 / 2359<br>66.0%<br>$p < 0.001$	1563 / 2495<br>62.6%<br>$p < 0.001$
LPN	513 / 717<br>71.5%<br>$p < 0.001$	1566 / 2326<br>67.3%<br>$p < 0.001$	628 / 1005<br>62.5%<br>$p < 0.001$	468 / 800<br>58.5%<br>$p < 0.001$
BPN	Not tested	656 / 1106<br>59.3%<br>$p < 0.001$	580 / 991<br>58.5%<br>$p < 0.001$	459 / 810<br>56.7%<br>$p < 0.001$
HPN	Not tested	414 / 646<br>64.1%<br>$p < 0.001$	438 / 691<br>63.4%<br>$p < 0.001$	365 / 593<br>61.6%<br>$p < 0.001$
CSS	Not tested	795 / 1159<br>68.6%<br>$p < 0.001$	335 / 556<br>60.3%<br>$p < 0.001$	333 / 553<br>60.2%<br>$p < 0.001$

Supplementary Table 1: Behavioural performance statistics

Observations	Degrees of Freedom	Chi^2 statistic vs. constant model	p-value vs. constant model
1913	1911	0.134	0.714

Supplementary Table 2: Comparison of ferret F1301 performance with full localization cues or with ITDs only (logistic regression)

Observations	Degrees of Freedom	Chi^2 statistic vs. constant model	p-value vs. constant model	
9082	9077	56.9	<0.001	
Comparisons:				
	Estimate	Standard Error	T-statistic	p-value
Intercept	0.890	0.036	25.079	<0.001
BBN:LPN	-0.167	0.057	-2.953	0.003
BBN:BPN	-0.513	0.071	-7.257	<0.001
BBN:HPN	-0.311	0.089	-3.483	<0.001
BBN:CSS	-0.109	0.073	-1.505	0.132

Supplementary Table 3: Comparison of ferret F1302 performance with full localization cues or with restricted cues or in the presence of a competing sound source (logistic regression)

Observations	Degrees of Freedom	Chi^2 statistic vs. constant model	p-value vs. constant model	
5602	5597	19.5	0.001	
Comparisons:				
	Estimate	Standard Error	T-statistic	p-value
Intercept	0.663	0.043	15.263	<0.001
BBN:LPN	-0.153	0.078	-1.955	0.051
BBN:BPN	-0.319	0.078	-4.102	<0.001
BBN:HPN	-0.115	0.090	-1.271	0.204
BBN:CSS	-0.247	0.097	-2.552	0.011

Supplementary Table 4: Comparison of ferret F1310 performance with full localization cues or with restricted cues or in the presence of a competing sound source (logistic regression)

Observations	Degrees of Freedom	Chi^2 statistic vs. constant model	p-value vs. constant model	
5251	5246	11.3	0.0235	
Comparisons:				
	Estimate	Standard Error	T-statistic	p-value
Intercept	0.517	0.041	12.493	<0.001
BBN:LPN	-0.174	0.083	-2.097	0.036
BBN:BPN	-0.249	0.082	-3.030	0.002
BBN:HPN	-0.046	0.094	-0.494	0.621
BBN:CSS	-0.103	0.096	-1.065	0.287

Supplementary Table 5: Comparison of ferret F1313 performance with full localization cues or with restricted cues or in the presence of a competing sound source (logistic regression)

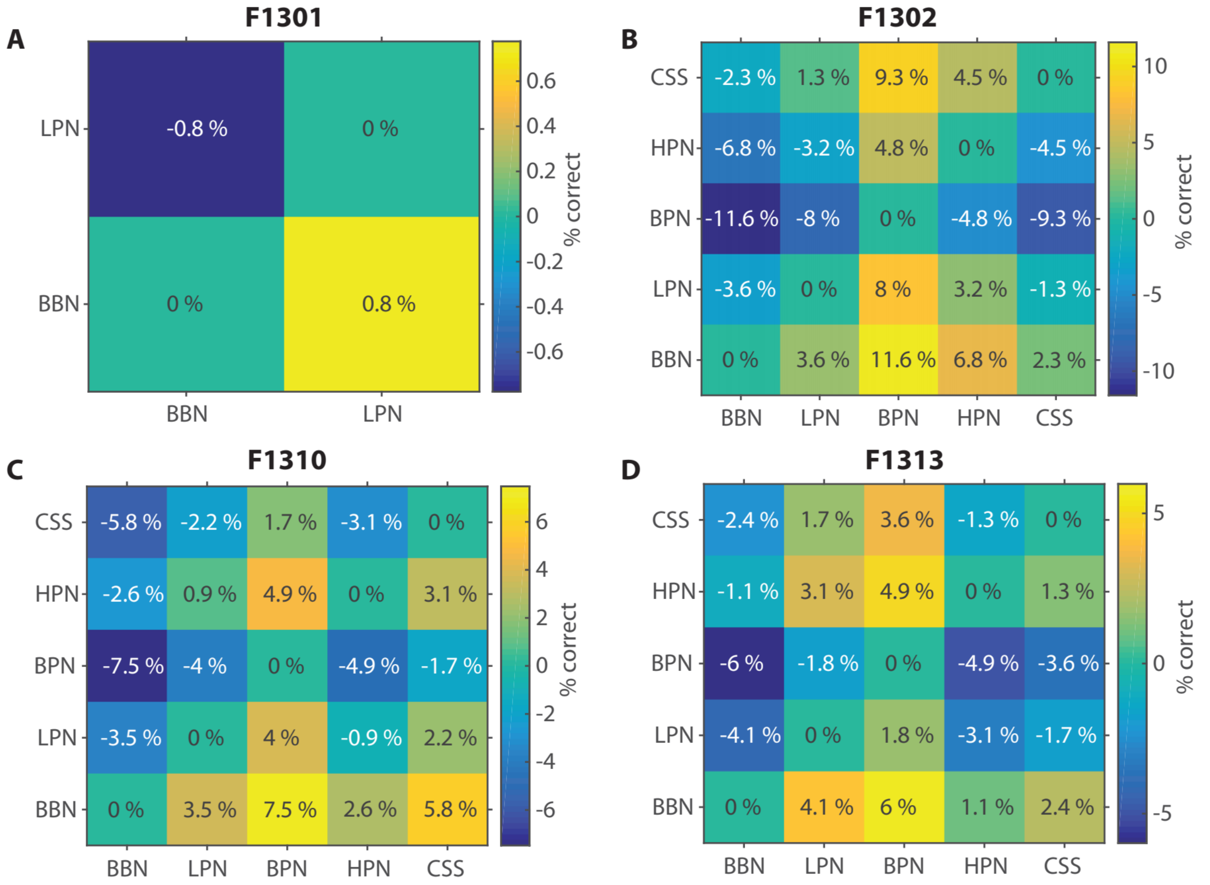

Supplementary Figure 1: Change in performance between stimulus conditions for each ferret. (A) Ferret: F1301, (B) F1302, (C) F1310, (D) F1313.

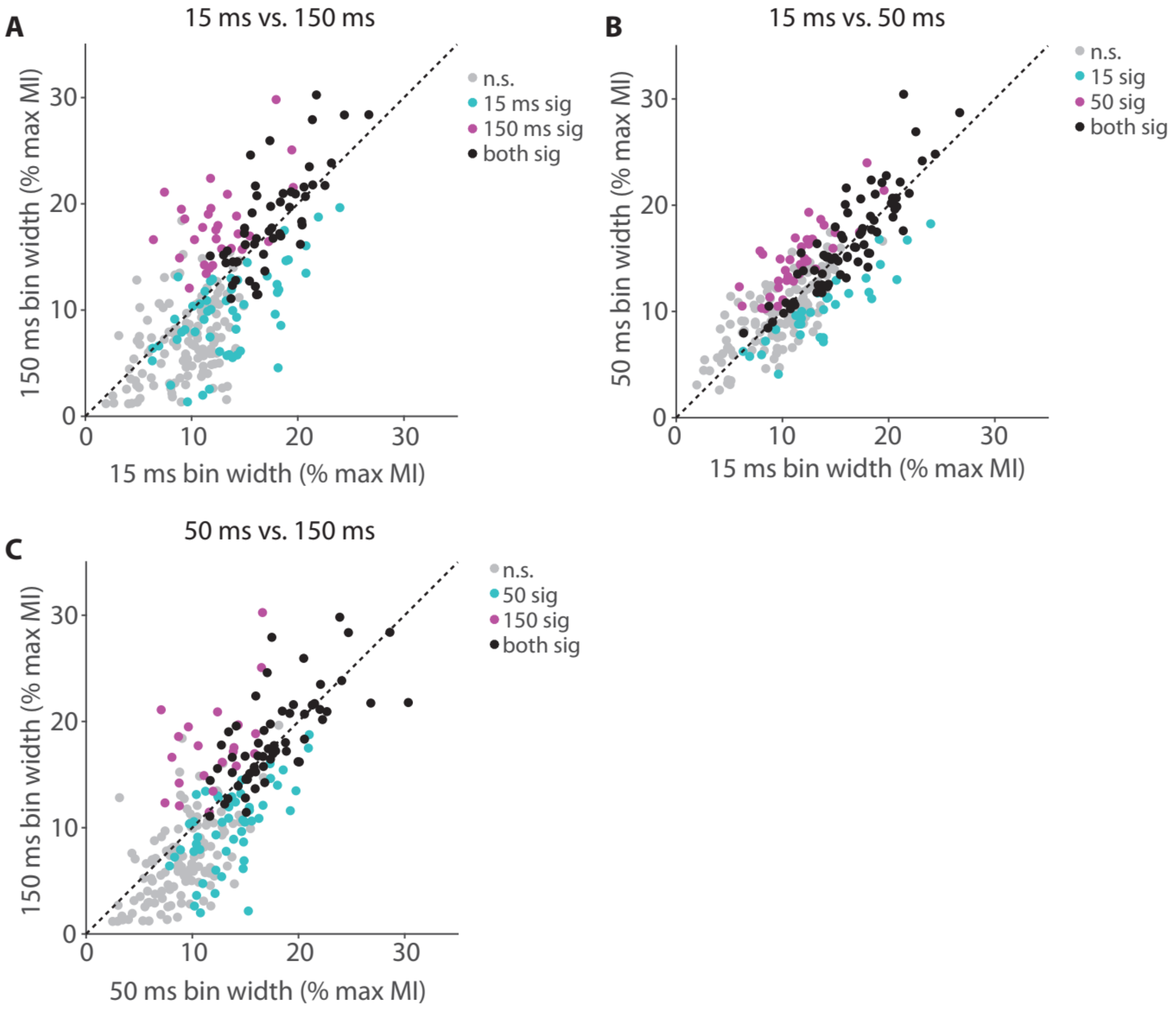

Supplementary Figure 2: Comparison of bin width on decoder performance for broadband stimuli. Black circles indicate units with significant MI in both decoder bin widths. Cyan and magenta circles indicate units with significant MI in a single decoder bin width. Grey circles indicate units with no significant MI in either bin width. For (A) 15 ms vs. 150 ms bin widths (black circles N = 70, cyan circles N = 31, magenta circles N = 37, grey circles N = 115), (B) 15 ms vs. 50 ms (N = 49, 52, 29, 123), (C) 50 vs. 150 ms (N = 57, 50, 21, 125).

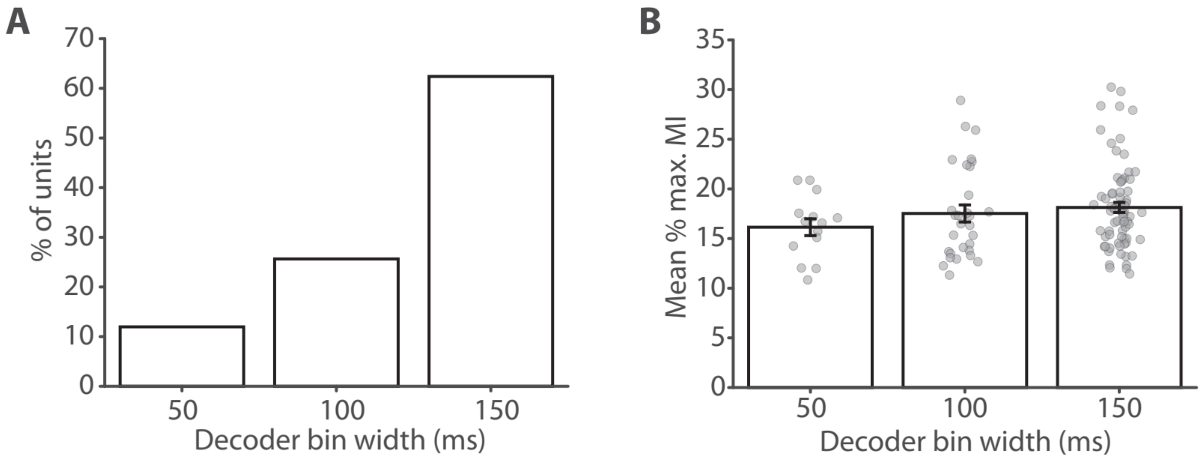

Supplementary Figure 3: Effect of increasing bin width on units with spatial information. (A) Shows the percentage of significantly informative units with the best decoding performance in 50, 100 or 150 ms bin widths (N = 117). (B) Percentage of maximum MI of from (A) in each decoder bin width, grey symbols indicate each cell. Statistics: one-way ANOVA Tukey-Kramer post-hoc pairwise comparisons.

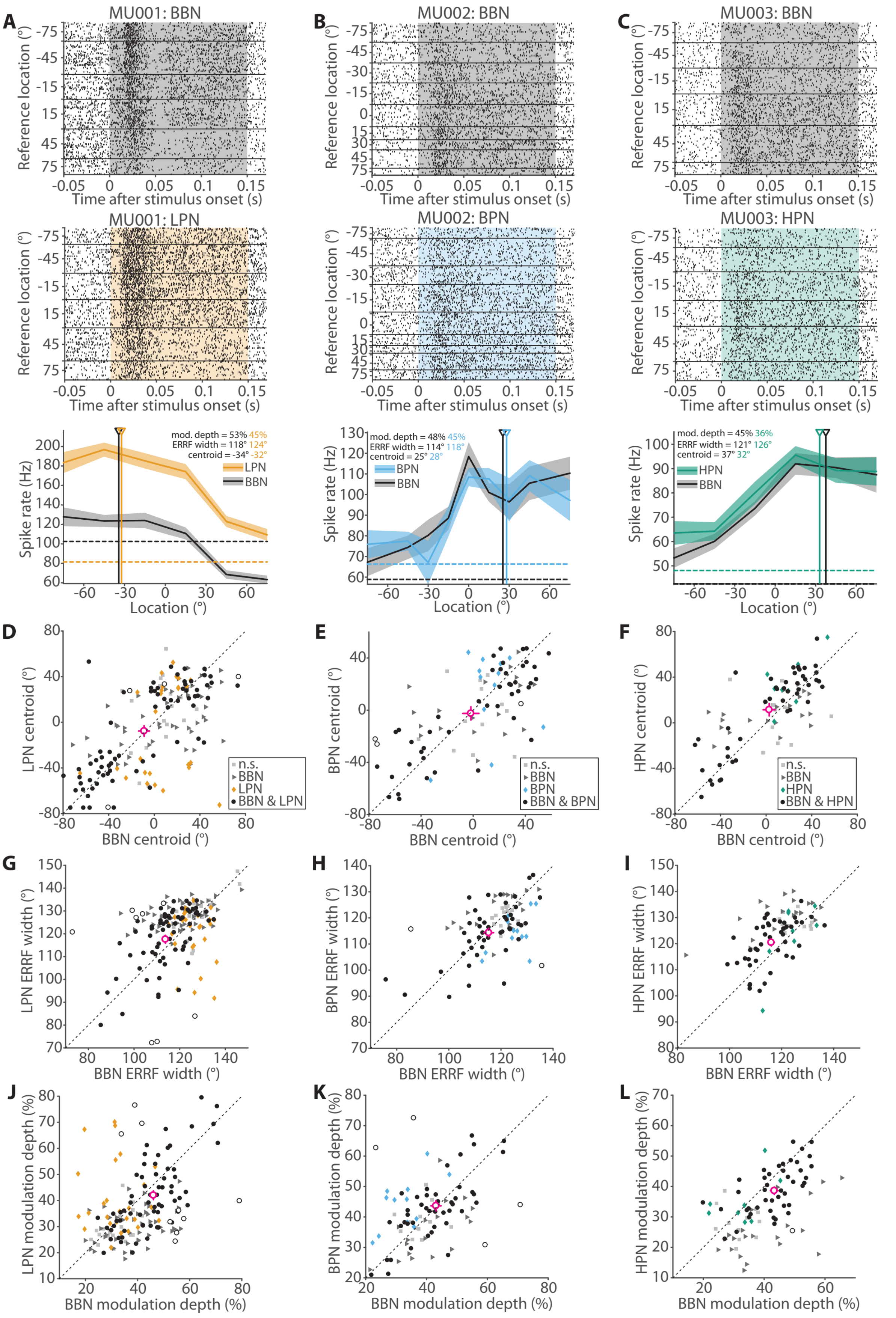
A
MU001: BBN
Reference location (°)
Time after stimulus onset (s)
MU001: LPN
mod. depth = 53% 45%
ERRF width = 118° 124°
centroid = -34° -32°
LPN
BBN
Spike rate (Hz)
Location (°)
B
MU002: BBN
MU002: BPN
mod. depth = 48% 45%
ERRF width = 114° 118°
centroid = 25° 28°
BPN
BBN
C
MU003: BBN
MU003: HPN
mod. depth = 45% 36%
ERRF width = 121° 126°
centroid = 37° 32°
HPN
BBN
D
LPN centroid (°)
BBN centroid (°)
n.s.
BBN
LPN
BBN & LPN
E
BPN centroid (°)
n.s.
BBN
BPN
BBN & BPN
F
HPN centroid (°)
n.s.
BBN
HPN
BBN & HPN
G
LPN ERRF width (°)
BBN ERRF width (°)
H
BPN ERRF width (°)
I
HPN ERRF width (°)
J
LPN modulation depth (%)
BBN modulation depth (%)
K
BPN modulation depth (%)
L
HPN modulation depth (%)

Supplementary Figure 4: Changing spectral content of the stimuli had little effect on spatial tuning properties of units. (A-C top row) Example raster from units in response to broadband (BBN) stimuli. (A-C middle row) rasters of responses to low-pass (LPN, A), band-pass (BPN, B) and high-pass (HPN, C) stimuli of the same units in the top row. (A-C bottom row) Spatial receptive fields of each unit in response to BBN and LPN (A), BBN and BPN (B), and BBN and HPN (C) stimuli. (D) Centroids of units recorded in both BBN and LPN conditions. Black circles show units spatially tuned in both conditions (N = 75), orange diamonds show units tuned to the LPN condition alone (N = 26), dark grey triangles show units tuned to BBN alone (N = 47) and grey shows units tuned in neither condition (N = 19). (E) Same as (D) for BBN and BPN comparison (N = 47, 12, 18, 12). (F) Same as (D) for BBN and HPN comparison (N = 45, 8, 21, 12). (G) shows comparisons of the ERRF widths for BBN and LPN. (H) shows the comparison of EERF width for BBN and BPN. (I) shows the comparison of ERRF widths for BBN and HPN. (J) shows the comparison of modulation depths for BBN and LPN. (K) shows the comparison of modulation depths for BBN and BPN. (L) shows the comparison of modulation depths for BBN and HPN. For (D-L) the mean $\pm$ s.e.m. of the jointly tuned units (black circles) is shown by crosshairs (circle, magenta). Open circles indicate individual units that significantly changed between stimuli.

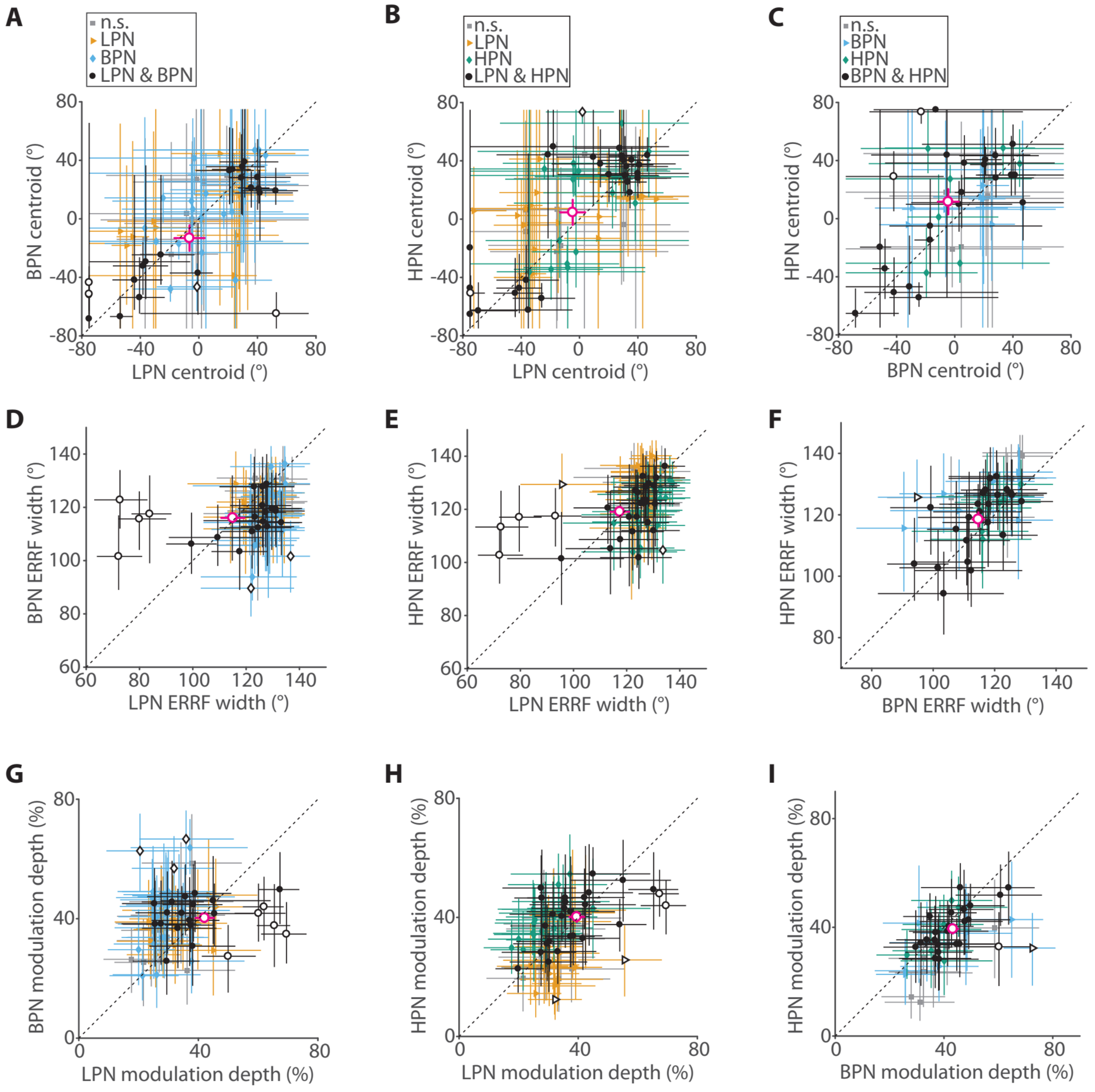

Supplementary Figure 5: Comparison of spatial tuning properties between cue-restricted stimuli. For all panels: Crosshairs show the 95% confidence intervals of each comparison. Units that were spatially tuned in both conditions (circles,), either condition alone (diamonds/triangles) or tuned in neither (squares). Mean ± s.e.m. of units spatially modulated in both conditions (i.e. of the black circles) is shown by the magenta circles and crosshairs. Open shapes indicate individual units that significantly changed between stimuli. (A) Centroid comparison between low-pass stimuli (LPN) and band-pass (BPN, both tuned N = 22, LPN tuned = 13, BPN tuned = 24, neither tuned = 11). (B) Centroid comparison between LPN and high-pass stimuli (HPN, both tuned N = 27, LPN tuned = 19, HPN tuned = 29, neither tuned = 10). (C) Centroid comparison between BPN and HPN (both tuned N = 23, BPN tuned = 9, HPN tuned = 7, neither tuned = 7). (D) ERRF width comparison between LPN and BPN. (E) ERRF width comparison between LPN and BPN. (F) ERRF width comparison between BPN and HPN. (G) Modulation depth comparison between LPN and BPN. (H) Modulation depth comparison between LPN and HPN. (I) Modulation depth comparison between BPN and HPN.

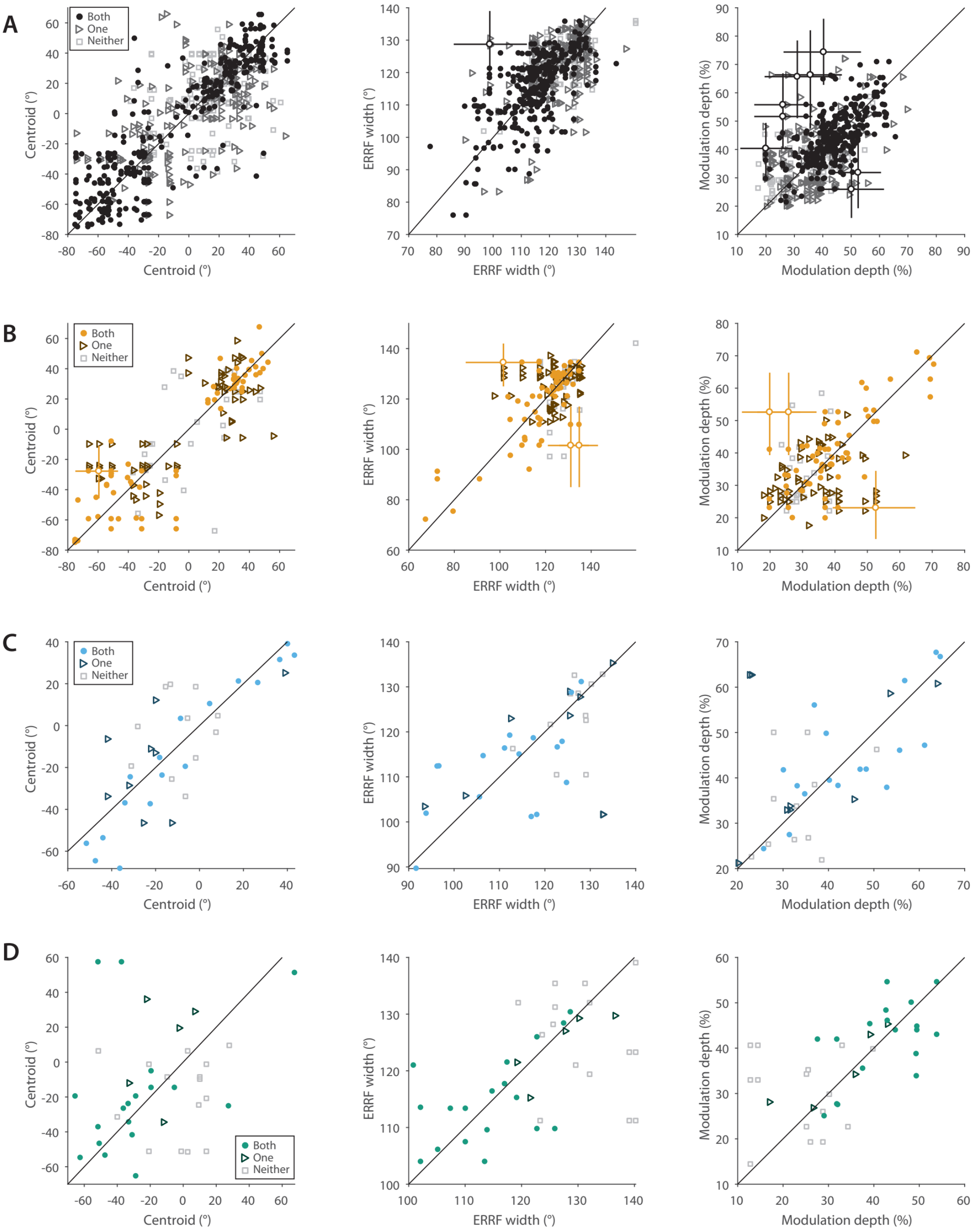

Supplementary Figure 6: Comparison of centroid, ERRF width and modulation depth of units recorded at the same site. Comparison of centroids (1st column), ERRF widths (2nd column) and modulation depths (3rd column) from units recorded at the same site but on different days in response to (A) broadband (BBN, number of cells, N = 149), (B) low-pass (LPN, N = 70), (C) band-pass (BPN, N = 32) and (D) high-pass (HPN, N = 22) stimuli. Units were either both spatially modulated (filled circles, *number of comparisons* for A-D *N* = 317, 65, 17, 18), one of the pair spatially modulated (triangles, A-D *N* = 189, 61, 9, 5) or neither spatially modulated (squares, A-D, *N* = 109, 23, 11, 15). Open circles show data from recordings where the parameter significantly changed between recording sessions with the cross hairs indicating the 95% confidence intervals.

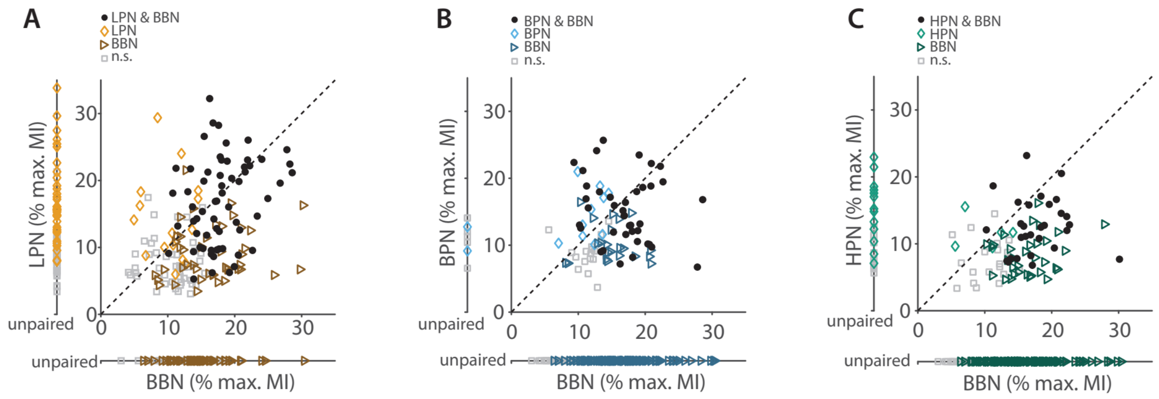

Supplementary Figure 7: Comparison of spatial information between full and cue-restricted stimuli. For all panels: Circles represent units with significant spatial information in both conditions, triangles represent units with information in BBN condition only, diamonds represent units with information in the limited cue condition (as indicated in the legends) and squares represent units with no information in either condition. Units that were only recorded in one condition are plotted along a separate axis (labelled unpaired). (A) Comparison of mean percentage of maximum MI in units recorded with both broadband (BBN) and low-pass (LPN) stimuli (Paired: both significant N = 62, LPN sig. = 15, BBN sig. = 44, neither sig. = 46. Unpaired: LPN sig. = 35, LPN not sig. = 42, BBN sig. = 47, BBN not sig. = 39). (B) Comparison of BBN and band-pass (BPN, Paired: both significant N = 40, BPN sig. = 10, BBN sig. = 22, neither sig. = 17. Unpaired: BPN sig. = 2, BPN not sig. = 6, BBN sig. = 91, BBN not sig. = 73). (C) Comparison of BBN and high-pass (HPN, Paired: both significant N = 29, HPN sig. = 5, BBN sig. = 33, neither sig. = 19. Unpaired: HPN sig. = 13, HPN not sig. = 15, BBN sig. = 91, BBN not sig. = 76).

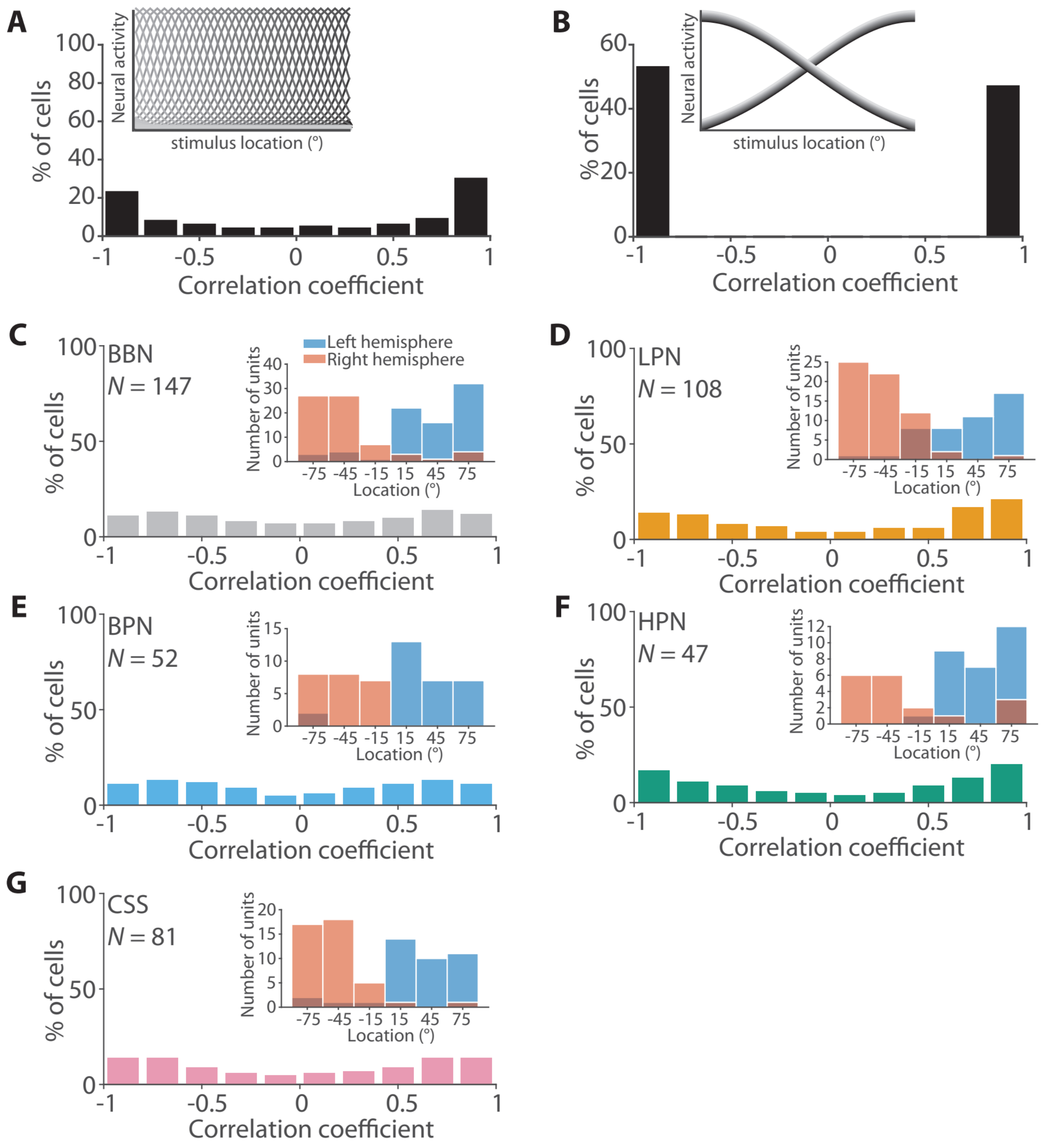

Supplementary Figure 8: Tuning curve correlations resemble those of a distributed model. Distribution of correlation coefficients for modelled (inset) distributed (A) and two-channel (B) tuning curves. (C-G) Distribution of correlation coefficients and best azimuths (inset) of tuning curves in each stimulus paradigm. Units had significant MI in at any bin width and were limited to those tested in the -75 to 75 testing locations so as to be comparable with performance in the population decoders (Figure 7).

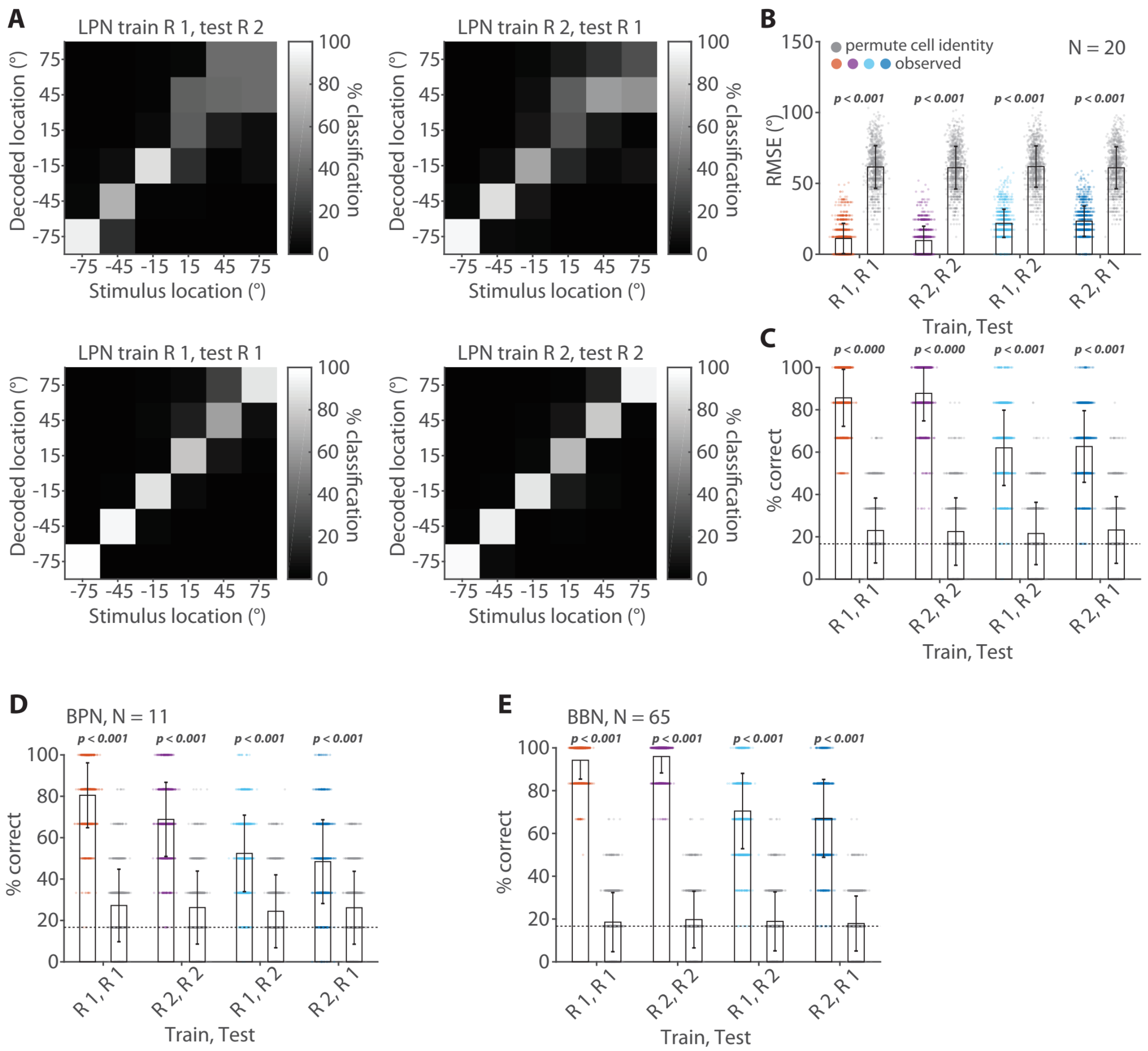

Supplementary Figure 9: Performance of the distributed decoder across recording sessions. The distributed decoder was trained and tested using responses of the same neurons to the same stimuli on different recording sessions. (A) Confusion matrices showing the % of decoded location classifications of each stimulus location when the distributed decoder was trained with responses in Recording 1 (R1) and tested with responses in Recording 2 (R2) to low-pass (LPN) stimuli from the same units that had significant spatial information in at least one bin width (top left), decoder trained on R2 responses and tested on R1 responses (top right), trained and tested on R1 (lower left) and trained and tested on R2 (lower right). (B) Root-mean squared error (RMSE) of the decoder (coloured symbols) compared with when the cell identities were shuffled (grey) in each pair of conditions. The bars show the mean ± std. dev. P values indicate the probability that the difference between the actual and shuffled mean RMSE is greater than that expected by chance (i.e. greater than the difference between real and shuffled means permuted 1000 times). (C) Mean ± std. dev. % correct of the decoder (coloured symbols) compared with % correct when cell identity was shuffled (grey). P values indicate the probability that the difference between the actual and shuffled mean % correct is greater than the difference obtained by chance. The black dotted lines indicates chance performance (1/6 chance of correct classification). (D) Same

as (C) but for responses to band-pass noise (BPN). (E) Same as (C) but for responses to broadband noise (BBN).

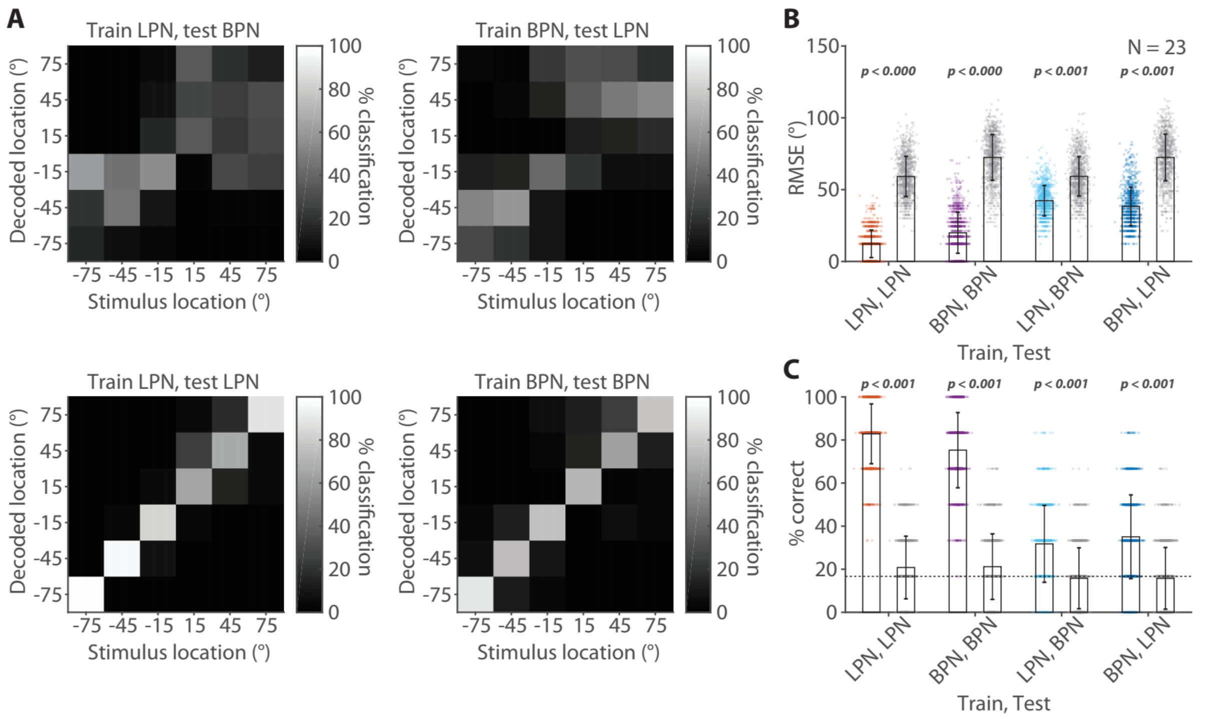

Supplementary Figure 10: Performance of the distributed decoder across spatial cues. The distributed decoder was trained and tested using responses of neurons to stimuli containing limited spatial cues. (A) Confusion matrices showing the % of decoded location classifications of each stimulus location when the distributed decoder was trained with responses to low-pass (LPN, ITDs only) stimuli and tested with responses to the band-pass (BPN, mainly ILDs and no fine-structure ITDs) stimuli from the same units that had significant spatial information in at least one bin width (top left), decoder trained on BPN responses and tested on LPN responses (top right), trained and tested on LPN (lower left) and trained and tested on BPN (lower right). (B) Root-mean squared error (RMSE) of the decoder (coloured symbols) compared with when the cell identities were shuffled (grey) in each pair of conditions. The bars show the mean ± std. dev. P values indicate the probability that the difference between the actual and shuffled mean RMSE is greater than the difference expected by chance (greater than the difference between real and shuffled means permuted 1000 times). (C) Mean ± std. dev. % correct of the decoder (coloured symbols) compared with % correct when cell identity was shuffled (grey). P values indicate the probability that the difference between the actual and shuffled mean % correct is greater than the difference obtained by chance. They black dotted line indicates chance performance (1/6 chance of correct classification).
