# Supplementary material for: Neurons in primary auditory cortex represent sound source location in a cue-invariant manner
Source: Nat Commun. 2019 Jul 9;10:3019. doi: 10.1038/s41467-019-10868-9 (PMC6616358; doi:10.1038/s41467-019-10868-9)
Supplement: Supplementary file 3 — Reporting Summary [file 41467_2019_10868_MOESM3_ESM.pdf]

nature research

Corresponding author(s): Katherine C. Wood, Jennifer K. Bizley

Last updated by author(s): Jun 5, 2019

Reporting Summary

Nature Research wishes to improve the reproducibility of the work that we publish. This form provides structure for consistency and transparency in reporting. For further information on Nature Research policies, see Authors & Referees and the Editorial Policy Checklist.

Statistics

For all statistical analyses, confirm that the following items are present in the figure legend, table legend, main text, or Methods section.

n/a	Confirmed	
☐	☒	The exact sample size (*n*) for each experimental group/condition, given as a discrete number and unit of measurement
☐	☒	A statement on whether measurements were taken from distinct samples or whether the same sample was measured repeatedly
☐	☒	The statistical test(s) used AND whether they are one- or two-sided<br>*Only common tests should be described solely by name; describe more complex techniques in the Methods section.*
☐	☒	A description of all covariates tested
☐	☒	A description of any assumptions or corrections, such as tests of normality and adjustment for multiple comparisons
☐	☒	A full description of the statistical parameters including central tendency (e.g. means) or other basic estimates (e.g. regression coefficient) AND variation (e.g. standard deviation) or associated estimates of uncertainty (e.g. confidence intervals)
☐	☒	For null hypothesis testing, the test statistic (e.g. *F*, *t*, *r*) with confidence intervals, effect sizes, degrees of freedom and *P* value noted<br>*Give P values as exact values whenever suitable.*
☒	☐	For Bayesian analysis, information on the choice of priors and Markov chain Monte Carlo settings
☒	☐	For hierarchical and complex designs, identification of the appropriate level for tests and full reporting of outcomes
☐	☒	Estimates of effect sizes (e.g. Cohen's *d*, Pearson's *r*), indicating how they were calculated

Our web collection on statistics for biologists contains articles on many of the points above.

Software and code

Policy information about availability of computer code

Data collection	Data was collected using Tucker-Davis-Technologies hardware and System 3 (OpenEx) software, combined with custom code written in Matlab (R2011b-2013b)
Data analysis	Data was analyzed using custom code written in Matlab (R2013b - R2017a)

For manuscripts utilizing custom algorithms or software that are central to the research but not yet described in published literature, software must be made available to editors/reviewers. We strongly encourage code deposition in a community repository (e.g. GitHub). See the Nature Research guidelines for submitting code & software for further information.

Data

Policy information about availability of data

All manuscripts must include a data availability statement. This statement should provide the following information, where applicable:

- Accession codes, unique identifiers, or web links for publicly available datasets
- A list of figures that have associated raw data
- A description of any restrictions on data availability

The datasets generated during and/or analysed in the current study are available from the corresponding authors on reasonable request. Data presented in all figures are available from figshare with the DOI: 10.6084/m9.figshare.c.4455089

Field-specific reporting

Please select the one below that is the best fit for your research. If you are not sure, read the appropriate sections before making your selection.

☒ Life sciences ☐ Behavioural & social sciences ☐ Ecological, evolutionary & environmental sciences

For a reference copy of the document with all sections, see nature.com/documents/nr-reporting-summary-flat.pdf

Life sciences study design

All studies must disclose on these points even when the disclosure is negative.

Sample size	The number of units recorded was determined by the success of electrode implantation. The number of trials performed for each stimulus condition was determined by the animal's behavior.
Data exclusions	No data were excluded from the study
Replication	Behavioral testing was replicated in four animals. Neurophysiological recording was repeated across units with the variability between units reported in the main text.
Randomization	Stimulus parameters on each trial were selected randomly from a uniform distribution without replacement.
Blinding	Data collection was blind to the behavioral performance on animals for specific stimulus parameters. Decoding analysis of neural data was blind to the identity of stimulus and behavioral features on tested trials.

Reporting for specific materials, systems and methods

We require information from authors about some types of materials, experimental systems and methods used in many studies. Here, indicate whether each material, system or method listed is relevant to your study. If you are not sure if a list item applies to your research, read the appropriate section before selecting a response.

Materials & experimental systems

n/a	Involved in the study	
☒	☐	Antibodies
☒	☐	Eukaryotic cell lines
☒	☐	Palaeontology
☐	☒	Animals and other organisms
☒	☐	Human research participants
☒	☐	Clinical data

Methods

n/a	Involved in the study	
☒	☐	ChIP-seq
☒	☐	Flow cytometry
☒	☐	MRI-based neuroimaging

Animals and other organisms

Policy information about studies involving animals; ARRIVE guidelines recommended for reporting animal research

Laboratory animals	Ferrets (Mustela putorius furo), Polecat variety, Female, 1-5 years
Wild animals	N/A
Field-collected samples	N/A
Ethics oversight	All animal procedures were approved by the local ethical review committee (University college London and Royal Veterinary College London Animal Welfare and Ethics Review Boards) and performed under license from the UK Home Office (Project License 70/7267) in accordance with the Animal (Scientific Procedures) Act 1986.

Note that full information on the approval of the study protocol must also be provided in the manuscript.
